# Basosquamous carcinoma: Comprehensive epidemiological, clinical, dermoscopic, and confocal features from a single center institution

**DOI:** 10.1111/srt.70012

**Published:** 2024-08-13

**Authors:** Federico Venturi, Valentina Erbacci, Giulia Veronesi, Biagio Scotti, Carlotta Baraldi, Emi Dika

**Affiliations:** ^1^ Oncologic Dermatology Unit IRCCS Azienda Ospedaliero‐Universitaria di Bologna Bologna Italy; ^2^ Department of Medical and Surgical Sciences (DIMEC) Alma Mater Studiorum University of Bologna Bologna Italy

**Keywords:** basosquamous carcinoma, dermoscopy, reflectance confocal microscopy, skin cancer

## Abstract

**Background:**

Basosquamous carcinoma (BSC) is a rare and aggressive nonmelanoma skin cancer (NMSC) that exhibits features of both BCC and squamous cell carcinoma (SCC). The gold standard for diagnosis is histopathological examination. BSC is often challenging to diagnose and manage due to its mixed histological features and potential for aggressive behavior

**Aim:**

To identify specific features aiding clinicians in differentiating BSCs using non‐invasive diagnostic techniques.

**Methods:**

We conducted a retrospective descriptive, monocentric study of the epidemiological clinical, dermoscopic, and reflectance confocal microscopy (RCM) features of histopathologically proven BSCs diagnosed between 2010 and 2023. A total of 192 cases were selected.

**Results:**

The study population consisted of 17 men (60.9%). Total 95.8% of patients at the time of diagnosis were ≥50 years. BSC occurred in the head and neck area in 124 cases (63.1%) of which 65 (33.9%) were in the H‐zone. For 47.4% of patients, BSC presented as a macule with undefined clinical margins (43.3%). Dermoscopic images were available for 98 cases: the most common parameter was the presence of whitish structureless areas (59 [60.2%]), keratin masses (58 [59.2%]), superficial scales, and ulceration or blood crusts (49 [50%] both). Vessels pattern analysis revealed hairpin vessels (exclusively) and linear irregular vessels as the most frequent (55 [56.1%] both). RCM examination was performed in 21 cases which revealed specific SCC features such as solar elastosis (19 [90.5%]), atypical honeycomb pattern (17 [89%]), proliferation of atypical keratinocytes (16 [80%]) combined with BCC’ ones as bright tumor islands (12 [57.8%]), and cleft‐like dark spaces (11 [53.4%]).

**Discussion:**

Our study reflects the largest cohort of BSCs from a single institution. We described an incidence rate of 4.7%, higher than reported in the Literature, with the involvement of patients ≥50years in almost 96% of cases and an overall male predominance. At clinical examination, BSC was described as a hyperkeratotic macule with undefined clinical margins with one or more dermoscopic SCC’ features, whereas the presence of typical BCC aspects was observed in less than 10% of cases, differently from what was previously reported. At RCM analysis, BSCs presented with an atypical honeycomb pattern with proliferation of atypical keratinocytes, hyperkeratosis, and in nearly 55% of patients, bright tumor islands with cleft‐like dark spaces.

**Conclusion:**

The distinctive dermoscopic patterns, along with the RCM features aid in the differentiation of BSCs from other NMSCs.

## 1 INTRODUCTION

Basosquamous carcinoma (BSC) is a rare and aggressive nonmelanoma skin cancer (NMSC) that exhibits features of both basal cell carcinoma (BCC) and squamous cell carcinoma (SCC).^1^ This malignancy is considered a subtype of NMSC and is characterized by the presence of both basaloid and squamous cell components within the tumor.^2^ BSC is often challenging to diagnose and manage due to its mixed histological features and potential for aggressive behavior. It may present as a nodular lesion, it may be ulcerated, displaying clinical, and dermoscopic features of both BCC and SCC. The gold standard for diagnosis is histopathological examination, with specific immunohistochemistry markers such as Ber‐EP4 and epithelial membrane antigen.^2^ Throughout the years, several studies described dermoscopic features of such tumors, to better understand this specific subtype of BCC and to make the diagnostic process easier.^3^ Recently, the introduction of noninvasive diagnostic tools such as reflectance confocal microscopy (RCM) allows high‐resolution imaging of the skin at a cellular level without the need for biopsy.^4^ Its application in NMSC has gained prominence both in the diagnostic and therapeutic setting, by giving a correct definition of presurgical margins, especially when tumors are located in highly sensitive areas.^5^ Thus, it may support the noninvasive recognition of BSC through the simultaneous detection of BCC‐associated feature and SCC‐associated feature. The use of RCM might be helpful also in the follow‐up surveillance for an early identification of tumor recurrences. To date, only one study has investigated such application.^6^ Due to its mixed composition, basosquamous carcinoma can exhibit more aggressive behavior than typical basal cell carcinomas, with an increased potential for invasion into deeper tissues and a higher risk of metastasis.^1^ Treatment usually involves surgical excision, Mohs micrographic surgery, or other therapeutic modalities, depending on the extent and location of the tumor.^7^, ^8^, ^9^, ^10^, ^11^, ^12^ Given the rarity and diagnostic complexities associated with basosquamous carcinoma, a multidisciplinary approach involving dermatologists, pathologists, and oncologists is mandatory for accurate diagnosis and effective management. Our retrospective analysis encompassed clinical, dermoscopic, RCM, and histopathologic characteristics of BSCs. Our objective was to identify specific features aiding clinicians in differentiating BSCs using non‐invasive diagnostic techniques.

## 2 METHODS

We conducted a retrospective descriptive, monocentric study of the epidemiological clinical, videodermoscopy (VDS), and RCM characteristics of histopathologically proven BSCs. We collected the histopathologic charts of BSCs diagnosed between 2010 and 2023 from the Laboratory of Dermatopathology, IRCCS of Sant'Orsola Malpighi Hospital. We obtained clinical and pathologic data from the charts for each patient. A total of 192 cases were selected. Patients were informed about the study and a written informed consent for publication of the photographs used in this manuscript was obtained. VDS images (20× and 40× magnification) were obtained using FotoFinderMedicam 800HD (FotoFinder, Germany) and were reviewed by three dermatologists with expertise in the field (F.V., G.V., and C.B.). Specific dermoscopic criteria previously validated were assessed: unfocused arborizing vessels, keratin masses, whitish structureless areas, superficial scales, ulceration or blood crusts, white structures (clods, circles, lines), blue‐grey blotches, blood spots in keratin mass, dotted vessels, polymorphous vascular pattern, pigmentation structures (brown dots, leaf‐like areas), focused arborizing vessels, linear irregular vessels (with dot vessels), hairpin vessels (exclusively).^3^ RCM investigation was performed with a Vivascope (MAVIG GmbH, Munich, Lucid‐Tech Inc., Henrietta, NY, USA) microscopy. Each skin lesions were systematically evaluated by RCM: one X–Y horizontal mapping (4 × 4‐mm mosaic) was performed at each epidermal layer beginning with the stratum corneum through the entire epidermis and until the papillary dermis (maximal depth of imaging: 200−250 µm). Lesion images were evaluated by two expert dermatologists in the field (F.V. and E.D.). We analyzed for each lesion the presence/absence of the most frequently described diagnostic RCM features for BCC (dark silhouettes, bright tumor islands, cleft‐like dark spaces, dendritic cells, plump‐bright cells, and canalicular vessels) and SCC (hyperkeratosis, parakeratosis, atypical keratinocytes, atypical honeycomb pattern of the spinous‐granular layer, solar elastosis, inflammatory infiltrate, blood vessel dilatation, and round nucleated cells).^13^, ^14^, ^15^

## 3 RESULTS

The study population consisted of 192 patients with a histologically confirmed diagnosis of BSC referred to the Oncologic Dermatology Unit of the IRCCS Azienda Ospedaliero‐Universitaria of Bologna between January 2010 and December 2023, including 117 men (60.9%) and 75 women (39.1%). A total of 95.8% of patients at the time of diagnosis were ≥50 years (range, 21–92 years). A total of 119 patients (62%) were between 70 and 90 years of age, and only 4.1% were younger than 50 years.

For the patients in this study, BSC occurred in the head and neck area (124 [63.1%] of which 65 [33.9%] in the H‐zone [periocular, nose, ear, mandibular area, and lips]), on the trunk (36 [18.7%]), and on the extremities (32 [16,7%]). For 47.4% of patients, BSC presented as a macule, for 25.3% as a papule, for 18.9% as a plaque, and as a nodule in 8.4% of cases. Among other clinical features, tumor margins were undefined in 43.3% of cases, hyperkeratosis was described in 32.6%, followed by ulceration (27.3%), pigmentation (22.1%), and erosion (15.9%). Dermoscopic and VDS images were available for 98 cases. Among them, the most common dermoscopic parameter was the presence of whitish structureless areas (59 [60.2%]), keratin masses (58 [59.2%]), superficial scales, and ulceration or blood crusts (49 [50%] both), blood spots in keratin mass (42 [42.8%]), white structures such as clods, circles and lines (35 [35.7%]), blue‐grey blotches (9 [9.2%]), and pigmentation structures such as brown dots and leaf‐like areas (8 [8.2%]). Vessels pattern analysis revealed hairpin vessels (exclusively) and linear irregular vessels as the most frequent (55 [56.1%] both), followed by polymorphous vascular pattern (53 [54%]), unfocused arborizing vessels (48 [49%]), focused arborizing vessels (11 [11.2%]), and dotted vessels (7 [7.1%]). RCM examination was performed in 21 cases. Specific BCC’ RCM features were described in half of the cases. Specifically, we described the presence of bright tumor islands (12 [57.8%]), cleft‐like dark spaces and canalicular vessels (11 [53.4%] both), dark silhouettes (9 [42.9%]), dendritic cells (2 [9.5%]), and plumb‐bright cells (5 [23.8%]). Among SCC features, we observed solar elastosis (19 [90.5%]), atypical honeycomb pattern of the spinous‐granular layer (17 [89%]), proliferation of atypical keratinocytes (16 [80%]), hyperkeratosis (12 [57.1%]), inflammatory infiltrate (11 [53.4%]), blood vessel dilatation (10 [47.6%]), round nucleated cells (6 [28.6%]), and parakeratosis (4 [19%]). Results are fully displayed in Table 1.

**TABLE 1:** Epidemiological, clinical, dermoscopic, and confocal features of basosquamous carcinoma.

Epidemiological data	N(192)	%
Sex		
Male	117	60.9
Female	75	39.1
Age at diagnosis		
≤30	1	0.5
30 ‐ 50	7	3.6
≥ 50	184	95.8
Tumor location		
Head and neck		
H‐zone	65	33.9
Extra H	59	30.7
Trunk	36	18.7
Extremities	32	16.7
**Clinical features**		
Elementary lesion		
Macule	45	47.4
Papule	24	25.3
Nodule	8	8.4
Plaque	18	18.9
Erosion	15	15.9
Ulceration	26	27.3
Hyperkeratosis	31	32.6
Pigmentation	21	22.1
Undefined tumor margins	41	43.2

Dermoscopic Features	**N (98)**	**%**
Unfocused arborizing vessels	48	49
Keratin masses	58	59.2
Whitish structureless areas	59	60.2
Superficial scales	49	50
Ulceration or blood crusts	49	50
White structures (clods, circles, lines)	35	35.7
Blue‐grey blotches	9	9.2
Blood spots in keratin mass	42	42.8
Dotted vessels	7	7.1
Polymorphous vascular pattern	53	54
Pigmentation structures (brown dots, leaf‐like areas)	8	8.2
Focused arborizing vessels	11	11.2
Linear irregular vessels (with dot vessels)	55	56.1
Hairpin vessels (exclusively)	55	56.1

**RCM features**	**N (21)**	**%**
BCC		
Dark silhouettes	9	42.9
Bright tumor islands	12	57.8
Cleft‐like dark spaces	11	53.4
Dendritic cells	2	9.5
Plump‐bright cells	5	23.8
Canalicular vessels	11	53.4
SCC		
Hyperkeratosis	12	57.8
Parakeratosis	4	19
Proliferation of atypical keratinocytes	16	80
Atypical honeycomb pattern of the spinous‐granular layer	17	89
Inflammatory infiltrate	11	53.4
Blood vessel dilatation	10	47.6
Solar elastosis	19	90.5
Round nucleated cells	6	28.6

## 4 DISCUSSION

**FIGURE 1:**
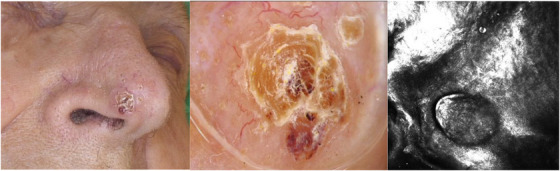
(A, B, C): Clinical, dermoscopic, and confocal presentation of basosquamous carcinoma of the nose. At clinical examination, an erythematous squamous nodule of the tip of the nose of a 77‐year‐old female patient (A). Dermoscopy revealed a white keratin mass in the center of the lesion with blood spots and peripheral arborizing vessels (B). RCM analysis at dermal–epidermal junction and superficial dermis showed basaloid cells with peripheral palisading and dark peritumoral clefts. Adjacent to those tumoral islands it was possible to observe larger, polygonal cells with dark nuclei and numerous bright, round cells corresponding to atypical keratinocytes and inflammatory infiltrate (C).

**FIGURE 2:**
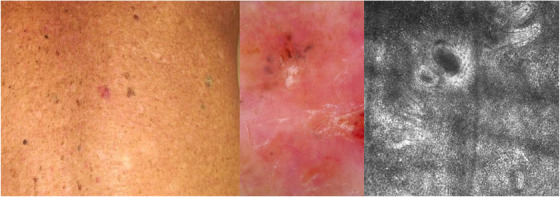
(A, B, C): Clinical, dermoscopic, and confocal presentation of basosquamous carcinoma of the back. At clinical examination, an erythematous macule located on the back of an 87‐year‐old patient (A). Dermoscopy revealed the presence of whitish structureless areas, superficial scales with blood spots in the keratin mass together with blue‐grey blotches in the upper part of the tumor on a background characterized by a polymorphous vascular pattern (B). RCM analysis showed an atypical honeycomb pattern, an inflammatory infiltrate with bright tumor islands and cleft‐like dark spaces (C).

BSC represents a challenging entity both for clinicians and pathologists due to its low incidence and atypical presentation. Our study, which reflect the largest cohort from a single institution, aimed to define such tumor with a comprehensive analysis of epidemiological, clinical, dermoscopic, and confocal features, in order to characterize BSCs and help clinicians in the diagnostic process. As far as we know, BSC is classified among BCC high‐risk subtype (WHO 2018), and is typified by clinical, dermoscopic, and histopathological features of both BCC and SCC.^1^, ^16^, ^17^, ^18^ In our center, we registered 192 cases of BSCs over a 13 year‐period, corresponding to 4.7% of all NMSCs. This data reflects a higher incidence than reported in the Literature, with an estimated incidence between 1.7 and 2.7%.^1^, ^19^ They occurred in almost 96% of cases in patients older than 50 years of age with an overall male predominance. The most common anatomical locations were the sun‐exposed areas of the head and neck (67% circa), more frequently in the H‐zone, interestingly. Given its more aggressive behavior if compared to other BCC subtype and this peculiar location in the high‐risk area of the face, a prompt recognition of such tumor using noninvasive diagnostic tools is mandatory to reduce misdiagnosis and facilitate clinicians in the management process. At clinical examination, BSC was described as a hyperkeratotic macule with undefined clinical margins in almost half of the cases (Figures 1A and 2A), differently from previous works that reported the typical presentation of such tumors as a nodule (vs. 8.4% of our series).^19^ Moreover, dermoscopic examination may help clinicians in the diagnostic process, and the detection of at least one BCC/SCC feature is suggestive of BSC (Figures 1B and 2B). In our series, most of the cases presented with one or more dermoscopic SCC’ features (whitish structureless areas, keratin masses, and superficial scales), whereas the presence of typical BCC aspects such as blue‐grey blotches and pigmented structures was observed in less than 10% of cases, differently from what was previously reported.^3^ Considering the vascular pattern, hairpin vessels were the most frequent observed together with linear irregular vessels, followed by a polymorphous vascular pattern and unfocused arborizing vessels. We further examined the presentation of BSCs at RCM, to define specific features and improve the diagnostic accuracy (Figures 1C and 2C). As far as we know, except from a single case report, this is the first study that analyze specific RCM features of such tumors.^6^ In our series, BSCs presented with an atypical honeycomb pattern of the spinous‐granular layer in almost 90% of cases, with proliferation of atypical keratinocytes, hyperkeratosis, and in nearly 55% of patients, bright tumor islands with cleft‐like dark spaces. Again, the diagnosis was confirmed at RCM when at least one specific BCC and SCC features was observed.

## 5 CONCLUSION

To conclude, our study provides valuable insights into the epidemiological, clinical, dermoscopic, and RCM features of BSCs based on the largest monocentric cohort. The distinctive dermoscopic patterns, along with the RCM features aid in the differentiation from other NMSCs. However, distinguishing BSCs remains a diagnostic challenge both for clinicians and pathologists, and larger studies are needed to confirm our results.

## CONFLICT OF INTEREST STATEMENT

The authors have no relevant financial or non‐financial interests to disclose.

## PATIENT CONSENT

Patients were informed about the use of their clinical information according to the Declaration of Helsinki principles and photos for publication intent. The informed consent was appropriately obtained during the medical examination.

## Data Availability

The data that support the findings of this study are available on request from the corresponding author. The data are not publicly available due to privacy or ethical restrictions.
